# Microaxial Left Ventricular Assist Device as Bridge to Transplantation in Postinfarction Left Ventricular Pseudoaneurysm

**DOI:** 10.1016/j.jaccas.2026.108011

**Published:** 2026-04-25

**Authors:** Sean Callahan, Nathan Horvat, Mingxi Yu

**Affiliations:** a Department of Internal Medicine, Loyola University Medical Center, Maywood, Illinois, USA; b Department of Cardiology, Loyola University Medical Center, Maywood, Illinois, USA

**Keywords:** acute heart failure, cardiac assist devices, inotropes, myocardial infarction, pseudoaneurysm

## Abstract

**Background:**

Left ventricular (LV) pseudoaneurysm is a rare complication of myocardial infarction (MI) that may present with cardiogenic shock.

**Case Summary:**

A 61-year-old man who had experienced MI 1 month prior presented with recurrent cardiogenic shock. Transthoracic echocardiography and cardiac magnetic resonance imaging revealed a large apical LV pseudoaneurysm compressing the right ventricle. Given prohibitive surgical risk, the patient was supported with prolonged Impella 5.5 therapy while awaiting heart transplantation. He remained hemodynamically stable throughout support and ultimately underwent successful heart transplantation.

**Discussion:**

Management of LV pseudoaneurysm typically involves surgical repair or conservative surveillance; however, literature describing prolonged mechanical circulatory support in this population remains sparse. This case demonstrates the feasibility of extended Impella support as a bridge to transplantation in a patient with post-MI LV pseudoaneurysm and cardiogenic shock. Impella-assisted LV unloading may mitigate aneurysmal wall stress, serving as a temporizing strategy in select high-risk patients.

**Take-Home Message:**

Prolonged Impella support may represent a viable bridge to heart transplantation in patients with LV pseudoaneurysm and cardiogenic shock who are unsuitable for surgical repair or durable left ventricular assist device therapy.

## Visual Summary

**Figure:**
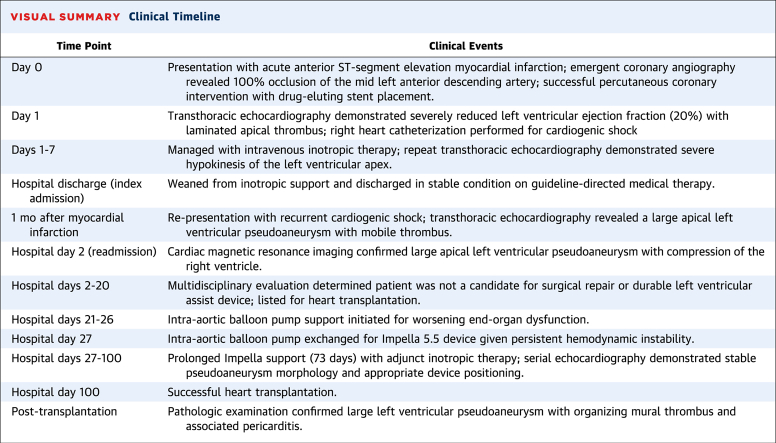

## History of Presentation

A 61-year-old man presented with acute chest pain and hypotension. He was found to have an anterior ST-segment elevation myocardial infarction and underwent emergent percutaneous coronary intervention of the left anterior descending artery (LAD). He was initiated on guideline-directed medical therapy and was discharged in stable condition.

**Take-Home Messages**

• Prolonged percutaneous Impella support can provide effective hemodynamic stabilization and serve as a bridge to heart transplantation in patients with post–myocardial infarction left ventricular pseudoaneurysm and cardiogenic shock who are not surgical candidates.
• Left ventricular unloading with Impella may reduce wall stress and mechanical tension on the pseudoaneurysm, potentially decreasing the risk of rupture during prolonged support.

One month later, the patient presented again with progressive dyspnea. Physical examination revealed hypotension (82/56 mm Hg), cool extremities, and elevated jugular venous pressure, concerning for cardiogenic shock.

## Past Medical History

The patient had a history of hypertension, hyperlipidemia, and coronary artery disease.

## Differential Diagnosis

Considerations included stent thrombosis, ventricular free wall rupture, left ventricular (LV) thrombus or pseudoaneurysm formation, acute mitral regurgitation, and ventricular septal defect.

## Investigations

**Figure 1:**
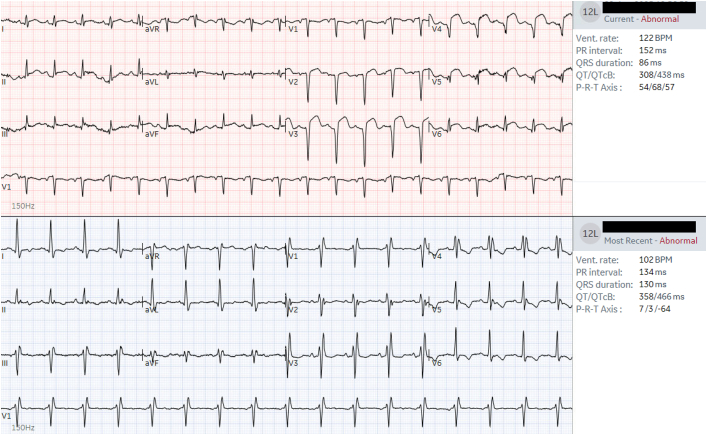
Electrocardiographic Findings at Presentation and Baseline 12-lead electrocardiograms demonstrating (Top) anterior ST-segment elevation myocardial infarction at presentation compared with (Bottom) a baseline electrocardiogram obtained approximately 1 year before.

During the first admission, the patient presented with acute chest pain, and the electrocardiogram demonstrated ST-segment elevations in the anterior leads (Figure 1). Emergent coronary angiography revealed a 100% occlusion of the mid-LAD and a 99% occlusion of the second obtuse marginal branch, for which a drug-eluting stent was successfully deployed in the LAD (Video 1). The angiogram favored the LAD as the culprit lesion. The second obtuse marginal branch, while demonstrating 99% subtotal occlusion, did not appear to supply a large enough territory to contribute significantly to the subsequent pseudoaneurysm formation.

Invasive hemodynamic assessment with right heart catheterization demonstrated a right atrial pressure of 7 mm Hg, pulmonary artery pressures of 28/20 mm Hg (mean: 21 mm Hg), a pulmonary capillary wedge pressure of 11 mm Hg, a pulmonary artery pulsatility index of 1.3, a cardiac output of 4.3 L/min, and a cardiac index of 1.95 L/min/m^2^. The gradient between pulmonary artery diastolic pressure and mean pulmonary capillary wedge pressure suggested a precapillary component of pulmonary hypertension.

Transthoracic echocardiography (TTE) performed 1 day after the infarction demonstrated a severely reduced LV ejection fraction of 20% with a laminated apical thrombus. The patient remained hospitalized on intravenous inotropic support and underwent repeat TTE 1 week after the myocardial infarction (MI), which demonstrated severe hypokinesis of the LV apex. He was subsequently weaned from inotropic support and was discharged in stable condition.

**Figure 2:**
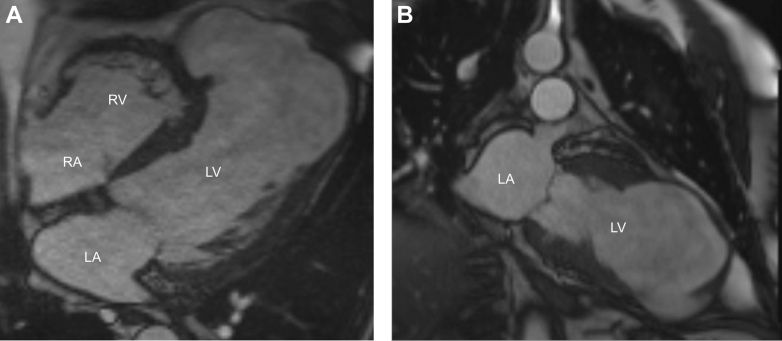
Cardiac Magnetic Resonance Imaging of Left Ventricular Pseudoaneurysm Cardiac magnetic resonance imaging demonstrating a large left ventricular pseudoaneurysm on (A) coronal and (B) sagittal views. The coronal image demonstrates right ventricular collapse due to mass effect from the pseudoaneurysm. LA = left atrium; LV = left ventricle; RA = right atrium; RV = right ventricle.

During the patient's second presentation 1 month later with recurrent cardiogenic shock, the admission TTE revealed interval development of a large apical pseudoaneurysm containing a mobile thrombus, with associated severe LV dilation (Video 2). Cardiac magnetic resonance imaging (CMR) performed the following day further characterized these findings and confirmed the presence of the apical pseudoaneurysm with associated right ventricular (RV) compression (Figure 2).

## Management

Upon readmission with cardiogenic shock, the patient was initially stabilized with intravenous inotropic therapy. Multidisciplinary evaluation deemed him unsuitable for surgical repair or durable left ventricular assist device (LVAD) implantation given the large pseudoaneurysm size and friable myocardium. He was listed for heart transplantation on hospital day 17.

**Figure 3:**
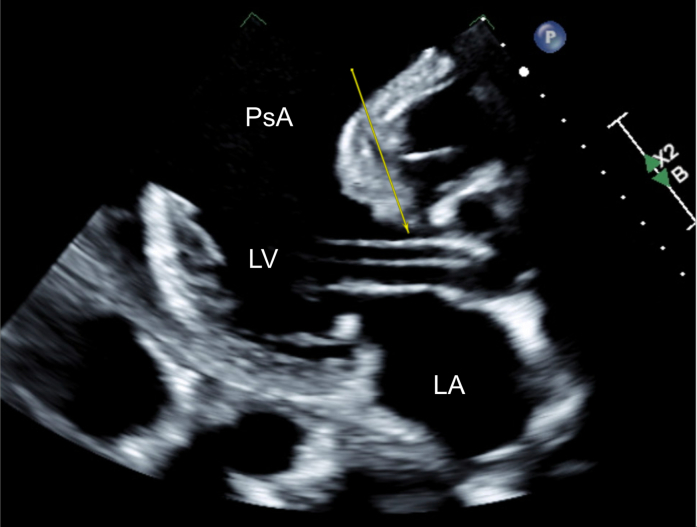
Transthoracic Echocardiography Demonstrating Impella Positioning Parasternal long-axis transthoracic echocardiographic view demonstrating the Impella device (yellow arrow) positioned within the left ventricle. A large left ventricular pseudoaneurysm is partially visualized. LA = left atrium; LV = left ventricle; PsA = pseudoaneurysm.

Intra-aortic balloon pump support was initiated from hospital days 21 to 26 for worsening renal and hepatic dysfunction. Given persistent hemodynamic instability, the intra-aortic balloon pump was exchanged for an Impella 5.5 device (Abiomed) on hospital day 27, providing effective LV unloading with improvement in systemic perfusion and cardiac output. The patient remained stable on Impella support (P-levels: 6-8) for 73 days with adjunct inotropic therapy along with inotropic therapy for additional RV support given evidence of RV dysfunction (low pulmonary artery pulsatility index on initial right heart catheterization and evidence of RV compression from pseudoaneurysm on prior TTE). Of note, the risk of thrombus aspiration was considered given LV apical thrombus identified on prior CMR, however preimplantation transesophageal echocardiography demonstrated interval resolution of thrombus, and the decision was made to proceed with Impella placement. Anticoagulation using a heparin drip was initiated, with target activated partial thromboplastin time range of 60 to 80 seconds while on Impella support. Serial echocardiography confirmed appropriate device positioning and stable pseudoaneurysm morphology throughout support (Figure 3). Notably, significant caution was exercised by both the primary and surgical teams during Impella repositioning to avoid disruption of the fragile pseudoaneurysm.

## Outcome and Follow-Up

The patient underwent successful heart transplantation on hospital day 100. Intraoperatively, severe pericarditis and dense adhesions between the endocardium and pericardium were noted near the pseudoaneurysm.

Pathologic examination of the explanted heart confirmed a large LV pseudoaneurysm containing organizing mural thrombus. Marked acute and organizing pericarditis was also present, consistent with chronic hemopericardium and prior surgical manipulation.

## Discussion

LV pseudoaneurysm is a rare complication of MI, occurring in 0.01% to 0.5% of cases, typically resulting from contained myocardial rupture.^1^ Presentation ranges from asymptomatic to fulminant cardiogenic shock. Diagnostic modalities including TTE, transesophageal echocardiography, CMR, and computed tomography are critical for distinguishing true aneurysms from pseudoaneurysms.^2^

Surgical repair is generally recommended for symptomatic or unstable pseudoaneurysms given high rupture risk, with reported in-hospital mortality approaching 20%.^3^ Long-term surgical survival is 73% at 1 year and 45% at 8 years.^3^ In contrast, asymptomatic patients may be managed conservatively, with 1-year survival of 88.9%; however, they remain at increased thromboembolic risk and often require long-term anticoagulation.^1^

While many reports describe surgical correction for unstable or symptomatic pseudoaneurysms,^4^^,^^5^ the literature describing prolonged mechanical circulatory support in this setting remains extremely limited. Our patient's pseudoaneurysm was exceptionally large, extending toward and compressing the right ventricle. Based on operative findings, the pseudoaneurysm likely became sealed and partially thrombosed, allowing the left ventricle to remain hemodynamically compensated with combined mechanical and pharmacologic support.

Prior reports include prolonged Impella support for a true LV aneurysm bridged to transplantation after 32 days^6^ and short-term Impella use preceding LVAD implantation in LV pseudoaneurysm.^7^ To our knowledge, this case represents one of the longest documented durations (>70 days) of Impella support in a patient with LV pseudoaneurysm before successful transplantation.

The Impella device unloads the LV by transferring blood from the LV into the ascending aorta, reducing wall stress and myocardial oxygen demand.^8^ In pseudoaneurysm physiology, unloading may further reduce mechanical tension on the pseudoaneurysm wall, decreasing risk of expansion or rupture.

Although current guidelines do not address percutaneous LV unloading in pseudoaneurysm management, this case suggests that prolonged Impella support may be a safe and effective bridge to transplantation when surgery or durable LVAD therapy is contraindicated.

## Conclusions

Impella support can provide durable hemodynamic stabilization and serve as an effective bridge to heart transplantation in patients with post-MI LV pseudoaneurysm and cardiogenic shock who are not surgical candidates.

## Funding Support and Author Disclosures

The authors have reported that they have no relationships relevant to the contents of this paper to disclose.

## Appendix

Video 1 Coronary Angiography Before and After Percutaneous Coronary Intervention Right anterior oblique views of left heart catheterization demonstrating (A) complete occlusion of the left anterior descending artery before intervention and (B) restoration of coronary flow after percutaneous coronary intervention with drug-eluting stent deployment in the setting of anterior ST-segment elevation myocardial infarction.

Video 2 Serial Echocardiographic Progression of Left Ventricular Pseudoaneurysm Transthoracic echocardiography in the apical 4-chamber view obtained at (A) 1 day, (B) 1 week, and (C) 1 month after myocardial infarction, demonstrating progressive enlargement of the left ventricular apical pseudoaneurysm.
